# Plant Growth-Promoting Fungi (PGPF) Instigate Plant Growth and Induce Disease Resistance in *Capsicum annuum* L. upon Infection with *Colletotrichum capsici* (Syd.) Butler & Bisby

**DOI:** 10.3390/biom10010041

**Published:** 2019-12-26

**Authors:** Banu Naziya, Mahadevamurthy Murali, Kestur Nagaraj Amruthesh

**Affiliations:** Applied Plant Pathology Laboratory, Department of Studies in Botany, University of Mysore, Mysuru 570 006, Karnataka, India; nazia@botany.uni-mysore.ac.in (B.N.); botany.murali@gmail.com (M.M.)

**Keywords:** chilli, *Colletotrichum capsici*, defense enzymes, host–pathogen interaction, induction of resistance, PGPF

## Abstract

In the current study, a total of 70 fungi were isolated from the rhizosphere soil of chilli collected from six different districts of south Karnataka, India. All the rhizospheric fungi were evaluated for its antagonistic nature against *Colletotrichum capsici*—the causal agent of anthracnose disease—and eight isolates were found positive. The antagonistic fungi were further characterized for the production of plant growth-promoting traits wherein five isolates were recorded positive for all the traits tested and were also positive for root colonization. All five plant growth-promoting fungi (PGPF) were subjected to molecular characterization for identification up to the species level and the accession numbers were obtained from NCBI. The five isolates, namely NBP-08, NBP-45, NBP-61, NBP-66 and NBP-67, were further experimented with on susceptible seeds to evaluate its efficacy on seed and plant growth parameters along with induction of resistance against the anthracnose disease. The evaluated PGPF offered significant enhancement in seed and plant growth parameters with maximum improvement observed in seeds treated with NBP-61. Among the PGPF isolates, NBP-61 showed the maximum protection of 78.75%, while all the other isolates also showed significant protection against anthracnose disease compared to untreated plants. The higher accumulation of lignin and callose deposition along with enhanced defense enzyme activities in the PGPF-treated challenge-inoculated seedlings authenticated the protection offered by PGPF. The study evidenced the immense ability of PGPF in eliciting disease protection and enhancement of plant growth in chilli, which may act as a possible substitute for harmful chemicals.

## 1. Introduction

Chilli (*Capsicum annuum* L.) is one of the important spices/vegetable crops cultivated all over the world in especially arid and semi-arid regions, for its fresh green and ripened fruits. Despite its importance in consumption, nutrition and medicine, chilli plants suffer from various diseases caused by bacteria, fungi, insects, etc. The anthracnose/dieback/fruit rot disease caused by *Colletotrichum capsici* (Sydow) E.J. Butler and Bisby (presently termed as *Colletotrichum truncatum*) is one of the most important biotic constraint of chilli as it affects the quality and quantity of the crop leading to a high yield loss of up to 84% [1,2]. The deciding factors for strong virulence of the pathogen are reduced availability of nutrients and environmental factors (like rainfall, temperature, humidity, etc.) [3]. Presently, the integrated disease management strategies used to combat anthracnose include biological, chemical and cultural practices that are employed individually or in combinations [2,4,5,6].

Abundant micro-organisms that co-exist in association with plant roots [plant growth-promoting fungi (PGPF) and rhizobacteria (PGPR)] have been effectively used in the induction of resistance in host plants against the invading phytopathogens apart from enhancing plant growth and development [7,8]. The application of these microorganisms is one of the eco-friendly disease management strategies that are long-lasting due to the induction of plants’ innate immunity [9]. To date, only a limited number of studies have reported on PGPF compared to PGPR to assess its efficacy in the induction of resistance against the invading pathogens compared to PGPR [10,11,12]. Plant growth-promoting fungi are non-pathogenic, naturally occurring saprophytes, which help to maintain soil fertility that, in turn, increases plant growth and induce frontline defense response against pathogen infections [10,12,13,14]. The root colonization ability of PGPF is considered to be the first and foremost mechanism involved in the prevention of pathogen infection and also helps in the uptake of nutrients, thereby enhancing plant growth [10,14,15]. The ability of the PGPF to solubilize phosphates and produce IAA, siderophore, cellulase, chitinase, etc., act directly or indirectly towards the enhancement of plant growth and development of a host, apart from inducing disease resistance [12,14,16].

Due to the profound beneficial effects of PGPF in agriculture, researchers have focused their attention on the application of PGPF for the induction of resistance and enhancement of plant growth by activating induced systemic resistance (ISR) in plants [14,15,16]. ISR by PGPF in plants involves the modification of cell walls by the accumulation of lignin, callose, phenols, etc., which primarily inhibit their entry and also prevent the growth and proliferation of the invading pathogen [12,17]. It is well documented that, apart from modification in the plant cell wall, PGPF is also known to activate enhanced accumulation of defense-related enzymes in plants (phenylalanine ammonia-lyase (PAL), peroxidases (POX), chitinase, β-1,3 glucanase, etc.) that are directly related to protective mechanisms against phytopathogens [10,11,18,19]. In the literature to date, there are no reports related to the application of PGPF in chilli for enhanced plant growth and disease resistance against anthracnose. Hence, a study was designed to assess the efficacy of PGPF isolated from the chilli rhizosphere in the soil to elicit plant growth promotion and induction of resistance against *C. capsici* for chilies grown under greenhouse conditions, and also to elucidate the possible mechanism of action involved at the histological and biochemical level.

## 2. Materials and Methods

### 2.1. Collection of Plant Material

Chilli seed samples cv. G4 susceptible to anthracnose disease (unpublished data) was procured from National Seed Corporation (NSC), Bengaluru, Karnataka, India, and used throughout the study.

### 2.2. Isolation and Identification of Microorganisms

#### 2.2.1. Pathogen

Anthracnose-infected chilli seed samples were collected from agricultural farms and subjected to isolation of *C. capsici* by SBM (standard blotter method) [20]. The collected seed samples were surface sterilized with a 0.2% sodium hypochlorite solution for a minute, followed by 2–3 washes with sterile distilled water (SDW). The sterilized seeds were placed on Petri dishes lined with autoclaved blotters (3 layers) moistened with SDW and were kept for incubation (25 ± 2 °C for 7 days). After incubation, the seeds were visualized under different magnifications of a stereomicroscope (Carl Zeiss, Discovery V20, Jena, Germany) and the colonies showing the typical sporulating structures of *C. capsici* were picked and inoculated on Petri plates containing potato dextrose agar (PDA) medium amended with chloramphenicol (200 µg L^−1^). The pathogen was identified based on their morphology, culture and conidial characters [21] (Figure S1). The genomic DNA was isolated from *C. capsici* following the cetyltrimethylammonium bromide (CTAB) method [22] and was subjected to amplification using ITS-1 (5′-TCCGTAGGTGAACCTGCGG-3′) and ITS-4 (5′- TCCTCCGCTTATTGATATGC-3′) [23] with initial denaturation at 95 °C (10 min), followed by denaturation for 35 cycles at 95 °C (1 min), annealing at 55 °C (1 min), primer extension at 72 °C (2 min) and final extension at 72 °C (8 min). The PCR-amplified products were electrophoretically detected on 1.5% (*w*/*v*) agarose gel containing 0.5 μg mL^−1^ of EB (ethidium bromide) and subjected for sequencing. The sequence was deposited in GenBank, NCBI, and accession number was acquired (Table S1).

#### 2.2.2. Rhizospheric Fungi

A total of 33 rhizosphere soil samples from the chilli-growing fields of Mysuru, Mandya, Chamarajanagara, Hassan, Ramanagara and Bengaluru districts, Karnataka, India, were collected. The collected rhizospheric soil samples were subjected to serial dilution for the isolation of rhizospheric fungi. About 1 mL of serially diluted soil samples (10^−4^ and 10^−5^) were inoculated by the spread plate method on Petri plates containing PDA medium amended with chloramphenicol (200 µg L^−1^). The inoculated fungi on PDA plates were incubated (25 ± 2 °C for seven days) and after incubation, the individual fungal colonies were aseptically picked and sub-cultured on PDA medium. Each of the isolated fungi was identified based on their morphology, culture and conidial characters [24]. Each isolate was named accordingly and used for further studies.

### 2.3. Evaluation of Rhizospheric Fungi for In Vitro Antagonism

All the isolated rhizosphere fungi were evaluated for their in vitro antagonism by the dual culture method against *C. capsici* [25]. The isolated rhizospheric fungi and *C. capsici* were inoculated on opposite sides in the Petri plates containing the PDA medium (1 cm apart from the edge of the plate) and incubated (25 ± 2 °C for seven days). The PDA plate inoculated with *C. capsici* alone served as control. After incubation, the radii of *C. capsici* towards and away from the rhizospheric fungi were measured and percent growth inhibition was calculated Equation (1):

(1) $$\mathrm{Inhibition}\;\mathrm{Percentage}\;\left(I\right)=\frac{R1-R2}{R1\;}\;\times \;100$$

where R1 is the colony radius of *C. capsici* in the control and R2 is the colony radius of *C. capsici* towards the antagonistic rhizosphere fungus.

### 2.4. Evaluation of Antagonistic Rhizospheric Fungi for Pathogenicity and Root Colonization in Chilli

Pathogenicity of rhizospheric fungal isolates was determined according to Koch’s postulates (https://phytopath.ca/wp-content/uploads/2014/09/What-are-Koch.pdf). The root colonization ability of selected rhizospheric fungi was evaluated according to Murali et al. [15] with modifications. The 15-day-old healthy chilli seedlings grown in autoclaved potting medium (soil, farmyard manure and sand in the ratio of 2:1:1) were drenched with 10 mL of each of the rhizospheric fungi (1 × 10^8^ CFU) separately (10 plants for each treatment) and maintained under greenhouse conditions for another 15 days. The 30-day-old plants were carefully uprooted and detached from the shoot without damaging. The detached roots were washed under a running tap and were fixed in 0.15% trichloroacetic acid [4:1 ethanol: chloroform (*v*/*v*)] for 10 min and stained with acid fuchsin (0.01%) and incubated for 10 min. After incubation, the stained root segments were observed under confocal microscopy (Carl Zeiss, Jena, Germany) excited at 568 nm and detected at 557 to 698 nm [26].

### 2.5. Evaluation of Antagonistic Rhizospheric Fungi for Plant Growth-Promoting (PGP) Traits

All the rhizosphere fungi that showed antagonism towards *C. capsici* growth were further assessed for their ability to produce plant growth-promoting properties in vitro.

#### 2.5.1. Qualitative Analysis

The halo zone formation around the rhizospheric fungi grown on Pikovskaya’s medium (PVK) [27], cellulose congo red agar medium [28], selective solid medium amended with colloidal chitin (4.5 g, *w*/*v*) [29] and chrome azurol S (CAS) agar medium [30] confirmed the solubilization ability of phosphate and production of cellulase, chitinase and siderophore, respectively. The indole acetic acid (IAA) production by rhizospheric fungi was validated by the development of pink colour in the culture filtrate of the potato dextrose broth (PDB) with added L-tryptophan (100 mg mL^−1^) [31]. The change in colour in the filter papers soaked with picric acid (0.5%) from yellow to light brown, which were placed on top of the test tubes inoculated with rhizospheric fungi on tryptic soy agar (TSA) media supplemented with glycine (4.4 g L^−1^), confirmed the production of hydrogen cyanide (HCN) [32].

#### 2.5.2. Quantitative Analysis

The selected rhizospheric fungi grown on PVK (phosphate solubilization), cellulose yeast extract (cellulase production), chitinase (chitinase production), CAS (siderophore production) and potato dextrose (amended with tryptophan (100 mg mL^−1^) (IAA production) broth for ten days were filtered using 0.45 μm Whatman No. 1 filter paper and centrifuged at 10,000 rpm for 10 min. The resultant culture filtrate (CF) was used for further studies.

Phosphate solubilization: The amount of phosphate solubilized in the CF was estimated by measuring the absorbance at 600 nm [33]. The concentration of phosphate (P) solubilized was determined against the standard graph of KH_2_PO_4_ and expressed in terms of percentage.

Cellulase production: The amount of cellulase produced by the degradation of cellulose into reducing sugars in the CF was estimated by measuring the absorbance at 540 nm and expressed as μmol of the reducing sugar released min^−1^ mg^−1^ protein [34].

Chitinase production: The amount of glucose released in the CF was determined by measuring the absorbance at 575 nm and calculated using the N-acetyl-D-glucosamine calibration curve [35] and expressed in nmol of glucose min^−1^ mg^−1^ protein.

Siderophore production: An equal volume of CAS reagent (60.5 mg L^−1^) was added to the CF and incubated for 20 min. After incubation, the absorbance of the sample was read at 630 nm and the percent siderophore solubilized was calculated according to Payne [36]. The amount of siderophore solubilized was expressed in percentage.

IAA production: To 1 mL of CF, double the volume of Salkowski reagent was added and incubated for 20 min. After incubation, the absorbance of the sample was read at 540 nm. The quantity of IAA produced was spectroscopically tabulated against the standard graph of IAA and expressed in terms of mg mL^−1^ [31].

HCN production: The filter paper in which a change in colour (yellow to light brown) was observed was eluted by placing them in 10 mL SDW for 10 min and the absorbance of the eluted solution was read at 625 nm [37] and expressed in OD values.

### 2.6. Molecular Identification of Plant Growth-Promoting Fungi (PGPF)

The rhizosphere fungi that were antagonistic to *C. capsici*, non-pathogenic and successfully colonized the chilli roots were further identified based on molecular characterization. The genomic DNA was isolated from all the selected fungi by the CTAB method and subjected to amplification using universal primers (ITS-1 and ITS-4) as mentioned above. The obtained amplified product was subjected to sequencing and the sequence was submitted to GenBank, NCBI, and accession numbers were acquired (Table S1).

### 2.7. Preparation of Inoculum

The 15-day-old culture of *C. capsici* grown on PDA media was flooded with 10 mL SDW and conidia were dislodged with the help of a sterile brush, aseptically. The resultant conidial suspension was passed through two layers of sterilized cheesecloth and the final concentration was adjusted to 5 × 10^5^ conidia mL^−1^ using SDW using Haemocytometer [38].

### 2.8. Preparation of Inducer and Seed Treatment

#### 2.8.1. Conidial Suspension of PGPF

The pure cultures of selected PGPF mass multiplied on PDA plates for 10–15 days were flooded with 10 mL of SDW and the conidia were dislodged with the help of a sterile brush, aseptically. The obtained conidial suspension was passed through two layers of sterilized cheesecloth and centrifuged. The resultant pellet was resuspended in SDW and the conidial suspension was adjusted to a final concentration of 1 × 10^8^ conidia mL^−1^ using SDW using Haemocytometer [39].

#### 2.8.2. Seed Treatment with PGPF

The surface-sterilized chilli seeds (cv. G4) were treated with conidial suspension by placing them in the conidial suspension of each of the PGPF (separately) and were kept in an incubator shaker (100 rpm at 25 °C) for 3 h and 6 h, respectively, to facilitate the penetration of the inducer. The PGPF-treated seeds were aseptically air-dried and used throughout the studies. The susceptible seeds treated with SDW served as control.

### 2.9. Evaluation of PGPF Seed Treatment on Seed Germination and Seedling Vigour of Chilli

The PGPF- and SDW-treated susceptible chilli seeds were placed equidistantly on moistened sterile blotter discs (3 layers) in Petri dishes to evaluate percent germination [20]. Another set of treated and untreated seeds were subjected to the between paper method to evaluate the seedling vigour [40]. All the treatments were incubated for fourteen days at 25 ± 2 °C and percent seed germination and seedling vigour were calculated Equations (2) and (3):

(2) $$\mathrm{Percent}\;\mathrm{Seed}\;\mathrm{Germination}=\frac{\;\mathrm{Total}\;\mathrm{number}\;\mathrm{of}\;\mathrm{seeds}\;\mathrm{germinated}}{\mathrm{Total}\;\mathrm{number}\;\mathrm{of}\;\mathrm{seeds}\;\mathrm{plated}}\times \;100$$

Vigour index = [Mean Root Length (cm) + Mean Shoot Length (cm)] × percent seed germination(3)

### 2.10. Evaluation of PGPF Seed Treatment on Plant Growth Parameters of Chilli

The plant growth parameters of PGPF- and SDW-treated chilli seeds were evaluated under greenhouse conditions in two sets for each treatment. The PGPF-treated and untreated susceptible chilli seeds (6 h) were sown in plastic pots (9 × 9 inch diameter) filled with autoclaved potting medium (soil, farmyard manure and sand in the ratio of 2:1:1) and were maintained under greenhouse conditions [25 ± 2 °C at 80% relative humidity (RH)]. The first set of plants were uprooted carefully, 45 days after sowing, to evaluate the vegetative growth parameters (plant height, fresh and dry shoot weight and total chlorophyll), while the other set of plants was used to determine the reproductive growth parameters (number of days required for flowering, number of fruits and fresh fruit weight), at 90 days after sowing the seeds.

### 2.11. Effect of PGPF Seed Treatment on Anthracnose Disease Resistance in Chilli under Greenhouse Conditions

The 30-day-old chilli plants raised under greenhouse conditions, as mentioned above upon treatment with PGPF and SDW, were subjected to a challenge inoculation with the conidial suspension of *C. capsici* (5 × 10^5^ conidial mL^−1^) till runoff. The challenge-inoculated plants were arranged in a randomized complete block design (RBD) and maintained under greenhouse conditions (25 ± 2 °C, 80% RH). At the end of the 30 days after the challenge inoculation, each plant was observed for the characteristic anthracnose symptoms and disease incidence was recorded. The percent disease index (PDI) was also calculated based on a 0–9 scale according to Mayee and Datar [41] with 0 as no symptoms and 9 representing lesions on leaves covering more than 51% Equations (4) and (5):

(4) $$\mathrm{Disease}\;\mathrm{Incidence}\;\left(\%\right)=\frac{\mathrm{Number}\;\mathrm{of}\;\mathrm{infected}\;\mathrm{plants}}{\mathrm{Total}\;\mathrm{number}\;\mathrm{of}\;\mathrm{plants}\;\mathrm{evaluated}}\times 100$$

(5) $$\mathrm{PDI}=\frac{\sum \;\left(\mathrm{Rating}\;\mathrm{number}\;\times \mathrm{No}.\mathrm{of}\;\mathrm{plants}\;\mathrm{with}\;\mathrm{the}\;\mathrm{rating}\right)}{\left(\mathrm{Total}\;\mathrm{No}.\mathrm{of}\;\mathrm{plants}\;\times \mathrm{Highest}\;\mathrm{rating}\right)}\times \;100$$

### 2.12. Effect of PGPF Seed Treatment on Histological and Biochemical Changes in Chilli

#### 2.12.1. Sampling of Seedlings

The PGPF- and SDW-treated susceptible chilli seeds (for 6 h) were subjected to the between paper method and incubated for 14 days at 25 ± 2 °C. After incubation, the seedlings were removed carefully (without damaging the roots) and root-dip inoculated with *C. capsici* (5 × 10^5^ conidia mL^−1^), while the control set was mock-inoculated with SDW. For histological studies, the seedlings were harvested at 0, 3, 6, 9, 12, 18 and 24 h after post-inoculation (h.p.i.) (immediately used), while for biochemical studies the seedlings were harvested 0, 3, 6, 12, 24, 48 and 72 h.p.i. and immediately stored at −80 °C until subsequent use.

#### 2.12.2. Histological Studies

Lignification: The epidermal peelings (from all the treatments) were kept in 2% phloroglucinol (dissolved in 95% ethanol) for 2 h, and after incubation they were mounted on a clean glass slide with a drop of 35% HCl. The slide was then heated over a low flame until a reddish-purple colour appears on the veins [42]. The peelings were immediately observed under the compound microscope (QUASMO, Haryana, India) and the percentage of lignified cells was calculated.

Callose deposition: The epidermal peelings (from all the treatments) were kept in 0.005% aniline blue [dissolved in 0.15 M dipotassium hydrogen phosphate buffer (pH 8.2)] for 1 h. After incubation, the peelings were mounted on a glass slide with 1–2 drops of glycerol [43] and observed under a fluorescence microscope (Carl Zeiss, Axio Imager A2, Jena, Germany) (k = 365 to 405 nm) to check the callose deposition and the percentage of callose-deposited cells were calculated.

#### 2.12.3. Biochemical Studies

Phenylalanine ammonia lyase (PAL) assay: One gram of chilli seedlings (from each treatment) were homogenized in 1 mL of 25 mM ice-cold Tris buffer (pH 8.8) containing 32 mM of β-mercaptoethanol in a prechilled mortar and pestle (separately). The homogenate was centrifuged (10,000 rpm for 25 min at 4 °C) and the resultant supernatant served as an enzyme source. To 0.5 mL of enzyme extract, 1 mL of 25 mMTris-HCl (pH 8.8) and 1.5 mL of 10 mM L-phenylalanine was added, thoroughly mixed and incubated for 2 h (40 °C). The reaction was stopped by adding 5 N HCl (0.18 mL) [44].

Peroxidase (POX) assay: One gram of chilli seedlings (from each treatment) was homogenized in 0.2 M sodium phosphate buffer (pH 6.5) in a pre-chilled mortar and pestle (separately). The homogenate was centrifuged at 10,000 rpm for 15 min at 4 °C and the resultant supernatant served as an enzyme source. The reaction mixture (3 mL) consisted of 0.25% (*v*/*v*) guaiacol in 10 mM potassium phosphate buffer (pH 6.9) containing 10 mM hydrogen peroxide. To the reaction mixture, 5 μL of enzyme extract was added to initiate the reaction and the absorbance was read at 470 nm [45].

β-1,3-glucanase assay: One gram of chilli seedlings (from each treatment) was homogenized in 0.05 M sodium acetate buffer (pH 5.2) in a pre-chilled mortar and pestle (separately). The homogenate was centrifuged (12,000 rpm for 15 min at 4 °C) and the resultant supernatant served as an enzyme source. To the enzyme extract (62.5 µL) an equal volume of 4% laminarin was added and incubated in a water bath (40 °C) for 10 min and the reaction was stopped by the addition of dinitro salicylic acid (375 µL) and kept on a water bath for 5 min. The absorbance of the reaction mixture was read at 540 nm [46].

Chitinase assay: One gram of chilli seedlings (from each treatment) were homogenized in 0.05 M sodium acetate buffer (pH 5.2) in a pre-chilled mortar and pestle (separately). The homogenate was centrifuged (10,000 rpm for 30 min at 4 °C) and the resultant supernatant served as enzyme source. To 300 µL of enzyme extract, an equal volume of substrate (colloidal chitin) was added and incubated at 37 °C for 2 h and the reaction was stopped by centrifugation. To 0.5 mL of supernatant, 0.1 mL of borate buffer (pH 9.8) was added and kept on a boiling water bath for 3 min and immediately cooled. To the reaction mixture, 1 mL of dimethylaminobenzaldehyde (DMAB) was added and incubated at 37 ± 2 °C for 20 min and the absorbance was read at 585 nm [47].

#### 2.12.4. Protein Estimation by Bradford Method

Protein content in chilli seedlings was estimated by the dye-binding method Bradford [48] using BSA (HiMedia) as a standard.

### 2.13. Statistical Analysis

The qualitative and quantitative analysis of plant growth-promoting properties and antagonistic studies were carried out in four replicates. The experiment on seed germination and seedling vigour consisted of four replications of 100 seeds each. The plant growth and disease protection studies consisted of 25 plants per treatment with four replicates in a randomized complete block design. The histological studies consisted of three replicates of 10 seedlings observed over 20 microscopic fields per treatment. The biochemical studies consisted of three replicates and were repeated thrice. The experimental data of the current study was subjected to Arcsine Transformation and Analysis of Variance (ANOVA) using SPSS v. 18.0 (SPSS Inc., Chicago, IL, USA). Significant effects of treatments were determined from the magnitude of the F-value (*p* ≤ 0.05). Treatment means were separated using Tukey’s HSD (honest significant differences) test.

## 3. Results

### 3.1. Isolation and Identification of Rhizospheric Fungi

From the chilli rhizosphere soil samples collected, a total of 70 fungi were isolated and each of them was identified based on the morphological, cultural and conidial characters down to the genus level. The fungal isolates belonged to nine different genera viz., *Alternaria*, *Aspergillus*, *Chaetomium*, *Curvularia*, *Fusarium*, *Melanaspora*, *Nigrospora*, *Penicillium* and *Trichoderma*. It was observed that among the genera, a maximum 18 isolates belonged to *Penicillium* sp. followed by *Aspergillus* sp. (16 isolates), while only one isolate of *Curvularia* sp. was isolated.

### 3.2. Evaluation of Rhizospheric Fungi for In Vitro Antagonism

All the isolated rhizospheric fungi were screened for antagonism towards the growth of *C. capsici* by dual culture assay. Among the isolates evaluated, a total of eight rhizosphere fungi were able to antagonize the mycelia growth of *C. capsici*, while other isolates had no antagonism towards the test pathogen (Table 1 and Table S2). A maximum percent inhibition of 88.64% on the growth of *C. capsici* was observed against *Talaromyces* sp. NBP-61, followed by *Penicillium* sp. NBP-45 (85.48%) and *Trichoderma* sp. NBP-67 (82.33%) (Figure S2). The rhizosphere fungi which were antagonistic to *C. capsici* were used for subsequent studies.

### 3.3. Evaluation of Antagonistic Rhizospheric Fungi for Pathogenicity and Root Colonization in Chilli Plants

The antagonistic rhizospheric fungi were further subjected to evaluate their pathogenicity and root colonization ability in chilli plants. Among the evaluated antagonistic rhizospheric fungi, six isolates were found non-pathogenic to chilli plants upon challenge inoculation, while only five isolates could colonize the roots (Table 1 and Figure S3). The rhizospheric isolates *Curvularia* sp. NBP-44 and *Fusarium* sp. NBP-65 were unable to colonize the roots and also produced necrotic spots (pathogenic symptoms) upon inoculation, while *Penicillium* sp. NBP-22 showed no pathogenic symptoms to the plant but was found negative towards root colonization.

### 3.4. Evaluation of Antagonistic Rhizospheric Fungi for Plant Growth-Promoting Traits

#### 3.4.1. Qualitative Analysis

The plant growth-promoting properties of all the eight antagonistic rhizosphere fungi evaluated are represented in Table 2 and Figure S4. Among the isolates evaluated, *Aspergillus* sp. NBP-08, *Penicillium* sp. NBP-22, *Penicillium* sp. NBP-45, *Talaromyces* sp. NBP-61, *Trichoderma* sp. NBP-66 and *Trichoderma* sp. NBP-67 showed a halo zone formation around the colony growth on selective media confirming the solubilization of phosphate and the production of cellulase, chitinase, and siderophore; they were also positive to the production of IAA and HCN. It was observed that, among the eight isolates, *Curvularia* sp. NBP-44 and *Fusarium* sp. NBP-65 was positive only for the production of IAA among the plant growth-promoting traits evaluated.

#### 3.4.2. Quantitative Analysis

The quantitative results were in accordance with the qualitative analysis of rhizosphere fungi. From the results of quantification of plant growth-promoting traits, it was observed that the phosphate solubilization ability was in the range of 43.38% to 64.74% and the production of IAA, siderophore, cellulase, chitinase and HCN was between 12.40 mg mL^−1^ and 65.45 mg mL^−1^, 59.37% and 89.27%, 0.35 U mg protein^−1^ and 0.92 U mg protein^−1^, 4.94 U mg protein^−1^ and 24.20 U mg protein^−1^ and 0.017 and 0.212 OD value, respectively (Table 2). Among the evaluated rhizosphere fungi, *Talaromyces* sp. NBP-61 possessed the higher quantifying ability of the evaluated traits compared to other isolates. Based on the results of root colonization, pathogenicity and the production of plant-growth-promoting properties, five rhizospheric isolates, namely *Aspergillus* sp. NBP-08, *Penicillium* sp. NBP-45, *Talaromyces* sp. NBP-61, *Trichoderma* sp. NBP-66 and *Trichoderma* sp. NBP-67 was designated as PGPF and used for further studies.

### 3.5. Evaluation of PGPF Seed Treatment on Seed Germination and Seedling Vigor of Chilli

The PGPF-treated and untreated chilli seeds (for 3 h and 6 h) were evaluated for their efficacy on seed growth parameters. The seed treatment with PGPF isolates was able to enhance seed germination and seedling vigour significantly (*p* ≤ 0.05) at both the time intervals evaluated compared to untreated control (Table 3). Among the PGPF assessed, a maximum of 86.25% and 1007.15 for seed germination and seedling vigour, respectively, was observed in chilli seeds upon treatment with *T. funiculosus* NBP-61 for 6 h followed by NBP-45 and NBP-08 treatments at the same time point. The SDW-treated seeds offered 70% seed germination and 197.17 for seedling vigour (Figure S5). From the results, it was noted that the PGPFs treatment to chilli seed for 6 h showed superior seed growth parameters compared to 3 h and subsequent studies were carried out in seeds treated with PGPF for 6 h.

### 3.6. Evaluation of PGPF Seed Treatment on Plant Growth Parameters

The PGPF-treated and untreated seeds were also evaluated for their effectiveness towards plant growth parameters. All the evaluated PGPFs significantly enhanced growth parameters upon treatment compared to control plants (Table 4). Amongst the PGPF treatments, maximum enhancement of vegetative and reproductive growth parameters were noticed in plants that were treated with NBP-61 with a maximum of 45.52 cm, 5.87 g plant^−1^, 3.65 g plant^−1^ and 36.51 mg g^−1^ of plant height, shoot fresh weight, shoot dry weight, shoot dry weight and total chlorophyll, respectively, and the plants flowered 10 days earlier compared to control plants with 28.5 fruits plant^−1^ with a weight of 4.22 g fruit^−1^. From the results, it was confirmed that the treatment of chilli seeds with PGPF positively enhanced plant growth parameters.

### 3.7. Effect of PGPF Seed Treatment on Anthracnose Disease Resistance in Chilli under Greenhouse Conditions

The effectiveness of seed treatment with PGPF against anthracnose disease resistance was carried out under greenhouse conditions. All the PGPF treatments offered significant disease protection towards anthracnose infection in chilli plants upon treatment compared to the control. Maximum protection of 78.75% was observed in plants raised upon the treatment NBP-61, followed by NBP-45 (69.75%) and NBP-08 (58.50%), respectively (Table 5). Amongst the different PGPF evaluated, seed treatment with NBP-61 offered 40.25% and 19% of DI and PDI, while in control plants 98.50% and 76% of DI and PDI, respectively, was observed.

### 3.8. Effect of Seed Treatment with PGPF on Histological and Biochemical Changes

#### 3.8.1. Histological Studies

**Figure 1:**
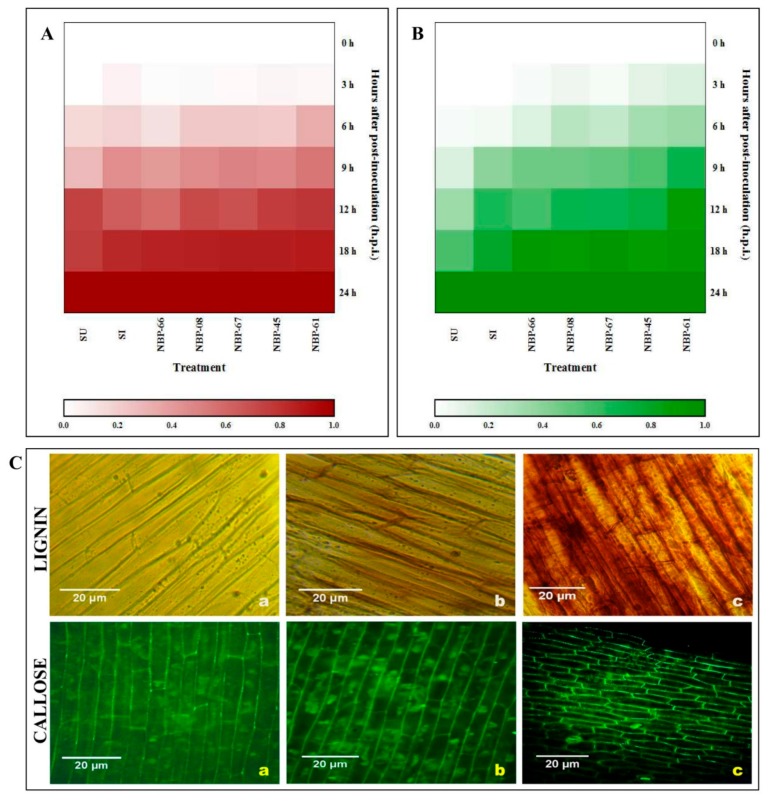
Lignin and callose deposition observed in the epidermal peelings of coleoptile regions of chilli seedlings treated with PGPF and challenge-inoculated with *C. capsici.* (**A**,**B**) Heat maps showing the pattern of the percentage of cells with lignin and callose deposition along the cell walls, respectively. (**C**) Representative light microscopic images showing the deposition of lignin and callose; a—susceptible uninoculated; b—susceptible inoculated; and c—NBP-61-treated and challenge-inoculated.

Lignification: The PGPF-treated and untreated chilli seedlings were evaluated for the deposition of lignin in their cell walls upon the challenge inoculation by differential staining. Change in the rapidity of lignification was noticed in susceptible, susceptible inoculated and PGPF-treated inoculated seedlings, with an increase in the time-interval of pathogen inoculation (Figure 1A,C). The formation of a reddish-brown colour along the cell walls confirmed lignification and the total number of cells with lignin deposition was recorded up to 24 h.p.i. Among the PGPF treatments, a maximum of 73.21% lignification was observed in cells treated with NBP-61, which was followed by 66.35% and 60.56% in the NBP-45 and NBP-08 treatment, respectively (Table S3). In susceptible challenge-inoculated seedlings, lignification was observed at 6 h.p.i. (4%) with a maximum of 29.55% at 24 h.p.i.

Callose deposition: Similar to lignification studies, callose deposition in cells of epidermal peelings were also observed in PGPF-treated and untreated seedlings upon a challenge inoculation by differential staining and observed under a fluorescence microscope (Figure 1B). The cells showing a bright greenish-yellow fluorescence was considered as positive for callose deposition (Figure 1C). The results were in accordance with that of lignin wherein the seedlings raised from NBP-61 showed the maximum percent (74.23% at 24 h.p.i.) of callose-deposited cells compared to all other treatments, including the control (Table S4). Callose depositions in cells of PGPF-treated inoculated seedlings were observed as early as 3 h.p.i., while in control challenge-inoculated seedlings, it was observed at 9 h.p.i. No significant deposition in callose was noticed up to 12 h.p.i. in susceptible challenge-inoculated seedlings.

#### 3.8.2. Biochemical Studies

**Figure 2:**
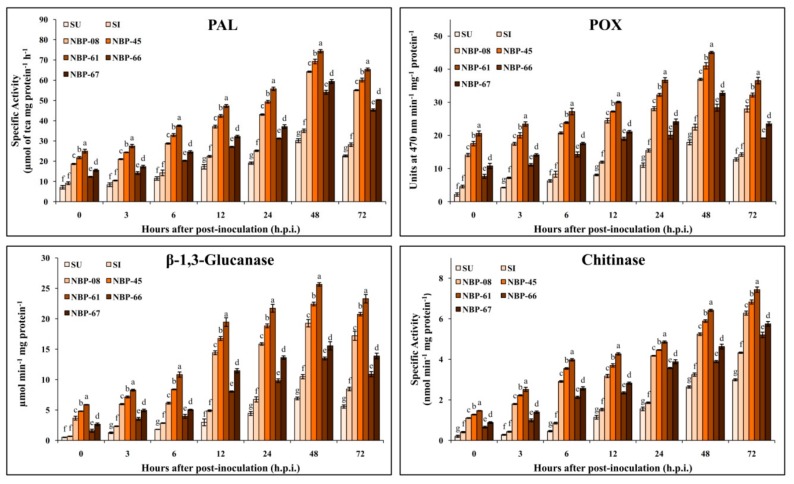
Effect of PGPF seed treatment on defense-related enzyme activity upon challenge inoculation with *C. capsici* in chilli.

Phenylalanine ammonia lyase (PAL) assay: The PAL enzyme activity increased progressively from 0 h.p.i. to reach a maximum at 48 h.p.i. in PGPF-treated challenge-inoculated seedlings and decreased at a later time point of evaluation (Figure 2). However, a maximum PAL activity of 74.35 U was noticed in chilli seedlings upon NBP-61 treatment at 48 h.p.i., followed by NBP-45 (69.27 U) and NBP-08 (64.22 U) treatments (Table S5). The susceptible inoculated and uninoculated seedlings showed a maximum enzyme activity of 35.04 U and 30.15 U, respectively, at 48 h.p.i. A total of 0.54 to a maximum of 1.12-fold increase in the enzyme activity was noticed between the PGPF-treated and susceptible seedlings upon challenge inoculation.

Peroxidase (POX) assay: Significant differences in POX enzyme activity was observed between PGPF-treated and untreated challenge-inoculated seedlings at each of the time intervals evaluated (Figure 2). Amongst the PGPF-treated challenge-inoculated seedlings, NBP-61 offered the highest POX activity of 45.06 U at 48 h.p.i., which was significant compared to other PGPF-treated seedlings (Table S6). There was a consistent enhancement in POX enzyme activity in all the PGPF treatments upon challenge inoculation that reached a maximum at 48 h.p.i. There was a 0.26- to 1-fold increase in the enzyme activity evaluated between PGPF-treated and susceptible inoculated seedlings upon the challenge inoculation.

β-1,3-glucanase assay: Progressive enhancement in β-1-3-glucanase activity was noticed from 3 h.p.i. to 48 h.p.i., irrespective of the samples evaluated upon challenge inoculation (Figure 2). A maximum of 25.64 µmol min^−1^ mg^−1^ protein of β-1-3-glucanase activity was observed in seedlings raised upon treatment with NBP-61, followed by 22.42 and 19.26 µmol min^−1^ mg^−1^ protein upon treatment with NBP-45- and NBP-08-treated seedlings, respectively. The susceptible challenge-inoculated and uninoculated seedlings offered 10.47 and 6.90 µmol min^−1^ mg^−1^ protein, respectively, at 48 h.p.i. An increase of 0.28- to 1.44-fold in enzyme activity was noticed in PGPF-treated challenge-inoculated seedlings compared to its respective control seedlings (Table S7).

Chitinase assay: The chitinase enzyme activity of the test samples at different time intervals is depicted in Figure 2 with the highest activity noticed at 72 h.p.i. in all the seedlings irrespective of the treatment. Among the PGPF-treated and challenge-inoculated seedlings, NBP-61-treated seedlings showed the highest chitinase activity of 7.43 nmol min^−1^ mg protein^−1^ (at 72 h.p.i.), which was significant (*p* ≤ 0.05) compared to other PGPF treatments and control seedlings (Table S8). The susceptible chilli seedlings with and without challenge inoculation offered 4.32 and 2.99 nmol min^−1^ mg protein^−1^ at 72 h.p.i., respectively. About a 0.2- to 0.71-fold increase in chitinase activity was observed in PGPF-treated and susceptible inoculated seedlings upon challenge inoculation.

## 4. Discussion

The microbes that are residents of rhizosphere soil (PGPF and PGPR) are one of the best suitable substitutes to enhance a plant’s innate immunity. Accordingly, all the rhizospheric fungi (70 isolates) were primarily screened for antagonism against *C. capsici*. The obtained results were in corroboration with other studies, wherein antagonistic studies were regarded as the primary criteria to select the positive isolates for their evaluation against disease resistance during host–pathogen interaction in many crop plants [10,49,50]. Further, the antagonistic nature exhibited by PGPF is attributed to hyper parasitism, antibiosis (synthesis of antibiotics), production of lytic enzymes or competition for space and nutrients, which directly help in the management of invading pathogens [12,15,49,50,51]. The qualitative analysis on the production of PGP traits confirmed that all the selected antagonistic isolates were positive for IAA, except NBP-44 and NBP-65, which were found negative for all the other traits evaluated. The quantitative analysis of PGP traits was in line with the qualitative results. Similarly, Vinayarani et al. [51] reported that rhizospheric fungi of turmeric were positive for PGP traits apart from possessing antagonistic activity against the invading pathogen and it is well attributed that the PGP-producing ability of the rhizosphere fungi is correlated to their non-pathogenic nature and disease suppression abilities [12,19].

The antagonistic rhizosphere fungi were further subjected to their pathogenic nature towards the host plant and the results confirmed that apart from NBP-44 and NBP-65, all were non-pathogenic to chilli and only five isolates (NBP-08, NBP-45, NBP-61, NBP-66 and NBP-67) possessed the root colonization ability. Likewise, Jogaiah et al. [16] have reported that the rhizosphere fungi that produced PGP traits were also non-pathogenic to host plants with very few offering pathogenicity. The antagonistic property of the rhizosphere fungi is asserted towards the offensive root colonization and defensive retention of rhizosphere niches that facilitate the production of siderophore, IAA and HCN, along with cell-wall degrading enzymes (cellulase and hemicellulase) that are reported to help in the improvement of plant growth and also induce resistance [13,14,49,51,52,53].

The susceptible chilli seeds upon treatment with PGPF offered a maximum of 86.25% and 1007.15 seed germination and seedling vigour, respectively, upon treatment with NBP-61. In addition, a significant increase in plant growth parameters was also observed upon PGPF seed treatment when compared to untreated plants with a maximum increase found in plants treated with NBP-61. Similarly, seed treatment with different PGPF, particularly of the genus *Aspergillus*, *Fusarium*, *Penicillium*, *Phoma* and *Trichoderma*, has been reported to improve seed and plant growth parameters in various agronomic and horticultural crops [10,14,54,55]. A maximum and minimum protection of 78.75% and 36.25% towards anthracnose infection were observed in plants raised upon the treatment of NBP-61 and NBP-66, respectively, which was significant compared to the untreated control plants. The current results are in accordance with the findings of other researchers, wherein seeds treated with a conidial suspension of PGPF resulted in enhanced disease protection against the invading pathogens in many crop plants [9,11,12,54]. The induction of resistance offered by PGPF is ascribed to the ability of PGP traits that, in turn, alter the metabolic pathways that help the host plants to defend against the invading pathogens [14,50].

The crossing of the plant cell wall is the primary challenge for pathogens to enter into the plant system and it serves as a source of signaling molecule in plants to trigger immune responses upon pathogen entry by deposition of defensive cell-wall materials, such as lignin, callose, phenol, etc. [56]. The deposition of lignin and callose along the cell walls is one of the primary modification processes employed by plants in response to pathogen attack [57,58]. A higher number of cells showed the callose and lignin accumulation along the cell walls in plants treated with PGPF upon challenge inoculation compared to its respective untreated control plants. In line with the results of the present study, higher accumulation of lignin and callose depositions in cell walls were also observed in PGPF-treated and challenge-inoculated cucumber plants, thereby forming a barrier for the entry of the invading pathogens [12,17].

The deposition of cell-wall materials, particularly lignin, is mainly regulated by defense-related enzymes (such as PAL, LOX, POX, PPO, chitinase, etc.) for lodging resistance in plants against invading pathogens [59]. PAL and POX enzymes play a requisite role in strengthening plant defense via structural deposition of lignin and phenolic compounds and also act as antagonistic to the invading pathogens by the production of phytoalexins (PAL), thereby hampering the growth and development of invading pathogen [55,59]. Similarly, the PGPF-treated chilli seedlings subjected to challenge inoculation showed the enhanced activity of PAL and POX enzymes compared to untreated challenge-inoculated seedlings, thereby authenticating the results obtained under histochemical studies. The results are also in line with many other studies wherein PGPF-treated and challenge-inoculated seedlings of cucumber, chilli, pearl millet and *Salvia* sp. offered higher activities of PAL and POX enzymes compared to control plants, thereby indicating their direct role in defense to host plants against pathogen attack [9,18,19]. Consequently, the enzymes β-1,3-glucanase and chitinase are also considered as direct defense enzymes of plants, which synergistically hamper pathogen growth and also enhance plant growth and reproduction [60]. Seed treatment with PGPF and upon challenge inoculation also resulted in enhanced activity of glucanase and chitinase enzymes, thereby inducing resistance in the host plants against pathogen infection [9,18]. It may be observed from the studies that seedlings treated with PGPF showed a similar pattern of enzyme activity irrespective of the PGPF treatment and enzyme studied. The results are well in agreement with our previous finding wherein a similar pattern of enzyme activity was observed irrespective of PGPF treatment and enzyme accumulation in pearl millet and cucumber [15,18]. The pattern of accumulation in enzyme activities may be attributed to the growth-promoting properties produced by the PGPF (like PGP traits), which exhibits the same set of defense-related activities during the host–pathogen interaction [16,19,51].

## 5. Conclusions

Overall, from the results it can be attributed that the PGPF isolated from the rhizospheric soil of chilli were able to enhance both seed and plant growth apart from inducing resistance to anthracnose disease. The mechanism of plant growth promotion and disease resistance offered by PGPF in the chilli–*C. capsici* host–pathogen interaction was further elucidated by histological and biochemical studies. Work is in progress to also assess the potential of *Talaromyces funiculosus* NBP-61 for the development of bio-formulations to use it as a bio-inoculant in the management of anthracnose disease in chilli.

## Figures and Tables

**Table 1:** In vitro antagonism, pathogenicity and root colonization ability of rhizosphere fungi.

Rhizospheric Fungi	* Antagonism (%)	^#^ Pathogenicity	^ Root Colonization
*Aspergillus* sp. NBP-08	77.91 ± 1.89 ^cd^	-	+
*Penicillium* sp. NBP-22	74.13 ± 0.63 ^d^	-	-
*Curvularia* sp. NBP-44	58.35 ± 1.62 ^e^	+	-
*Penicillium* sp. NBP-45	85.48 ± 0.63 ^ab^	-	+
*Talaromyces* sp. NBP-61	88.64 ± 0.72 ^a^	-	+
*Fusarium* sp. NBP-65	51.41 ± 1.20 ^f^	+	-
*Trichoderma* sp. NBP-66	79.81 ± 1.03 ^c^	-	+
*Trichoderma* sp. NBP-67	82.33 ± 1.03 ^bc^	-	+

Values are means of four independent replicates (n = 4) and ± indicate standard errors. Mean values followed by the same letter(s) within the same column are not significantly (*p* ≤ 0.05) different according to Tukey’s HSD. * Antagonistic to *C. capsici*; ^#^ Pathogenic to chilli plants; ^ Colonization of PGPF on chilli roots upon treatment. “+” indicates positive and “-” indicates negative for the experiments.

**Table 2:** Qualitative and quantitative analysis for plant growth-promoting (PGP) properties of antagonistic rhizosphere fungi.

Rhizospheric Fungi	Qualitative Analysis						Quantitative Analysis					
	PS	IAA	SID	CEL	CHI	HCN	PS (%)	IAA (mg mL^−1^)	SID (%)	CEL (min^−1^ mg^−1^ Protein)	CHI (min^−1^ mg^−1^ Protein)	HCN (O.D.)
*Aspergillus* sp. NBP-08	+	+	+	+	+	+	53.65 ± 0.02 ^c^	43.88 ± 0.75 ^c^	78.26 ± 0.44 ^c^	0.630 ± 0.025 ^c^	09.58 ± 0.02 ^c^	0.107 ± 0.004 ^c^
*Penicillium* sp. NBP-22	+	+	+	+	+	+	43.38 ± 0.61 ^f^	19.07 ± 0.97 ^f^	59.37 ± 0.97 ^f^	0.350 ± 0.007 ^f^	04.94 ± 0.04 ^f^	0.017 ± 0.004 ^e^
*Curvularia* sp. NBP-44	-	+	-	-	-	-	-	14.20 ± 1.17 ^g^	-	-	-	-
*Penicillium* sp. NBP-45	+	+	+	+	+	+	58.81 ± 0.27 ^b^	50.51 ± 0.95 ^b^	84.68 ± 0.35 ^b^	0.724 ± 0.024 ^b^	15.74 ± 0.12 ^b^	0.150 ± 0.004 ^b^
*Talaromyces* sp. NBP-61	+	+	+	+	+	+	64.74 ± 0.16 ^a^	65.45 ± 0.52 ^a^	89.27 ± 0.09 ^a^	0.921 ± 0.021 ^a^	24.20 ± 0.12 ^a^	0.212 ± 0.013 ^a^
*Fusarium* sp. NBP-65	-	+	-	-	-	-	-	12.40 ± 0.93 ^g^	-	-	-	-
*Trichoderma* sp. NBP-66	+	+	+	+	+	+	46.71± 0.22 ^e^	24.21 ± 0.98 ^e^	64.40 ± 0.92 ^e^	0.441 ± 0.005 ^e^	06.53 ± 0.08 ^e^	0.037 ± 0.004 ^e^
*Trichoderma* sp. NBP-67	+	+	+	+	+	+	51.56 ± 0.02 ^d^	34.68 ± 0.95 ^d^	73.96 ± 0.62 ^d^	0.525 ± 0.013 ^d^	08.00 ± 0.06 ^d^	0.070 ± 0.004 ^d^

Values are means of four independent replicates (*n* = 4) and ± indicate standard errors. Mean values followed by the same letter(s) within the same column are not significantly (*p* ≤ 0.05) different according to Tukey’s HSD. “+” indicates positive and “-” indicates negative for the experiment carried out. PS: phosphate solubilization; IAA: indoleacetic acid; SID: siderophore; CEL: cellulase; CHI: chitinase; HCN: hydrogen cyanide; “+” indicates positive and “-” indicates negative for the experiments.

**Table 3:** Plant growth-promoting fungi (PGPF) seed treatment on seed germination and seedling vigour of chilli.

Treatments	Hours	Seed Germination (%)	Seedling Vigour
Control	3 h	70.00 ± 0.40 ^f^	194.32 ± 4.67 ^i^
	6 h	70.00 ± 0.40 ^f^	197.17 ± 2.18 ^i^
NBP-08	3 h	79.00 ± 0.40 ^c^	571.50 ± 9.49 ^e^
	6 h	79.25 ± 0.47 ^c^	642.45 ± 7.36 ^d^
NBP-45	3 h	82.50 ± 0.64 ^b^	784.07. ± 2.85 ^c^
	6 h	83.00 ± 0.81 ^b^	813.82 ± 4.93 ^b^
NBP-61	3 h	86.00 ± 0.40 ^a^	830.40 ± 0.96 ^b^
	6 h	86.25 ± 0.47 ^a^	1007.15 ± 7.97 ^a^
NBP-66	3 h	73.00 ± 0.40 ^e^	358.05 ± 3.32 ^h^
	6 h	73.25 ± 0.62 ^e^	425.35 ± 4.09 ^g^
NBP-67	3 h	76.00 ± 0.40 ^d^	492.62 ± 8.57 ^f^
	6 h	76.25 ± 0.47 ^d^	569.65 ± 7.75 ^e^

Values are means of four independent replicates (*n* = 4) and ± indicate standard errors. Mean values followed by the same letter(s) within the same column are not significantly (*p* ≤ 0.05) different according to Tukey’s HSD.

**Table 4:** Effect of PGPF seed treatment on plant growth parameters of chilli under greenhouse conditions.

Treatments	Vegetative Growth Parameters				Reproductive Growth Parameters		
	Plant Height (cm)	SFW (g)	SDW (g)	Total Chlorophyll (mg g^−1^)	No. of Days for Flowering	No. of Fruits Plant^−1^	Fruit Weight (g)
Control	15.75 ± 0.69 ^f^	1.77 ± 0.08 ^f^	0.97 ± 0.04 ^f^	13.97 ± 0.37 ^f^	79.00 ± 0.40 ^d^	5.00 ± 0.40 ^f^	1.33 ± 0.02 ^f^
NBP-08	34.27 ± 0.49 ^c^	4.27 ± 0.04 ^c^	2.80 ± 0.07 ^c^	23.77 ± 0.13 ^c^	72.50 ± 0.64 ^b^	19.25± 0.47 ^c^	2.75± 0.13 ^c^
NBP-45	39.50 ± 0.64 ^b^	4.82 ± 0.13 ^b^	3.25 ± 0.11 ^b^	29.59 ± 0.54 ^b^	70.75 ± 0.47 ^ab^	24.25± 0.47 ^b^	3.50 ± 0.04 ^b^
NBP-61	45.52 ± 1.25 ^a^	5.87 ± 0.07 ^a^	3.65± 0.06 ^a^	36.51± 0.66 ^a^	68.50 ± 0.64 ^a^	28.50± 0.64 ^a^	4.22 ± 0.08 ^a^
NBP-66	21.87 ±1.00 ^e^	2.85 ± 0.06 ^e^	1.70 ± 0.04 ^e^	17.46 ± 0.11 ^e^	77.50 ± 0.86 ^cd^	10.00 ± 0.40 ^e^	1.70 ± 0.04 ^e^
NBP-67	30.32 ± 0.78 ^d^	3.40 ± 0.09 ^d^	2.12 ± 0.04 ^d^	21.30 ± 0.08 ^d^	75.50 ± 0.64 ^c^	14.25 ± 0.62 ^d^	2.32 ± 0.04 ^d^

Values are means of four independent replicates (*n* = 4) and ± indicate standard errors. Mean values followed by the same letter(s) within the same column are not significantly (*p* ≤ 0.05) different according to Tukey’s HSD.

**Table 5:** Effect of PGPF seed treatment on the induction of disease resistance in chilli against anthracnose disease.

Treatments	Disease Incidence(%)	Disease Protection (%)	No. of Infected Plants at Each Level						Percent Disease Index (PDI)
			0	1	3	5	7	9	
Control	98.50 ± 0.64 ^f^	0.00 ± 0.00 ^f^	02.00 ± 1.15	3.00 ± 1.00	3.00 ± 1.00	06.00 ± 1.15	66.00 ± 1.15	20.00 ± 1.63	76.00 ± 0.79 ^f^
NBP-08	49.25 ± 0.47 ^c^	58.50± 0.64 ^c^	37.00 ± 1.00	21.00 ± 1.91	17.00 ± 1.00	12.00 ± 1.63	10.00 ± 1.15	03.00 ± 1.00	25.44 ± 0.49 ^c^
NBP-45	45.25± 0.47 ^b^	69.75± 0.75 ^b^	49.00 ± 1.91	17.00 ± 1.00	10.00 ± 1.15	11.00 ± 1.91	10.00 ± 2.58	03.00 ± 1.00	22.11 ± 0.27 ^b^
NBP-61	40.25± 0.85 ^a^	78.75 ± 0.47 ^a^	53.00 ± 1.91	14.00 ± 1.15	16.00 ± 1.63	06.00 ± 1.15	10.00 ± 1.15	01.00 ± 1.00	19.00 ± 1.06 ^a^
NBP-66	67.25 ± 0.85 ^e^	36.25± 0.62 ^e^	18.00 ± 1.15	18.00 ± 1.15	30.00 ± 1.15	23.00 ± 1.91	08.00 ± 1.63	03.00 ± 1.00	34.00 ± 0.46 ^e^
NBP-67	63.25± 0.62 ^d^	47.50± 1.04 ^d^	28.00 ± 1.63	19.00 ± 1.91	24.00 ± 1.63	17.00 ± 1.91	10.00 ± 1.15	02.00 ± 1.15	29.33 ± 0.70 ^d^

Values are means of four independent replicates (*n* = 4) and ± indicate standard errors. Mean values followed by the same letter(s) within the same column are not significantly (*p* ≤ 0.05) different according to Tukey’s HSD.

## Supplementary Materials

The supplementary materials are available online at https://www.mdpi.com/2218-273X/10/1/41/s1.
