# Supplementary material for: Plant Growth-Promoting Fungi (PGPF) Instigate Plant Growth and Induce Disease Resistance in Capsicum annuum L. upon Infection with Colletotrichum capsici (Syd.) Butler & Bisby
Source: Biomolecules. 2019 Dec 26;10(1):41. doi: 10.3390/biom10010041 (PMC7023450; doi:10.3390/biom10010041)
Supplement: Supplementary file 1 [file biomolecules-10-00041-s001.pdf]

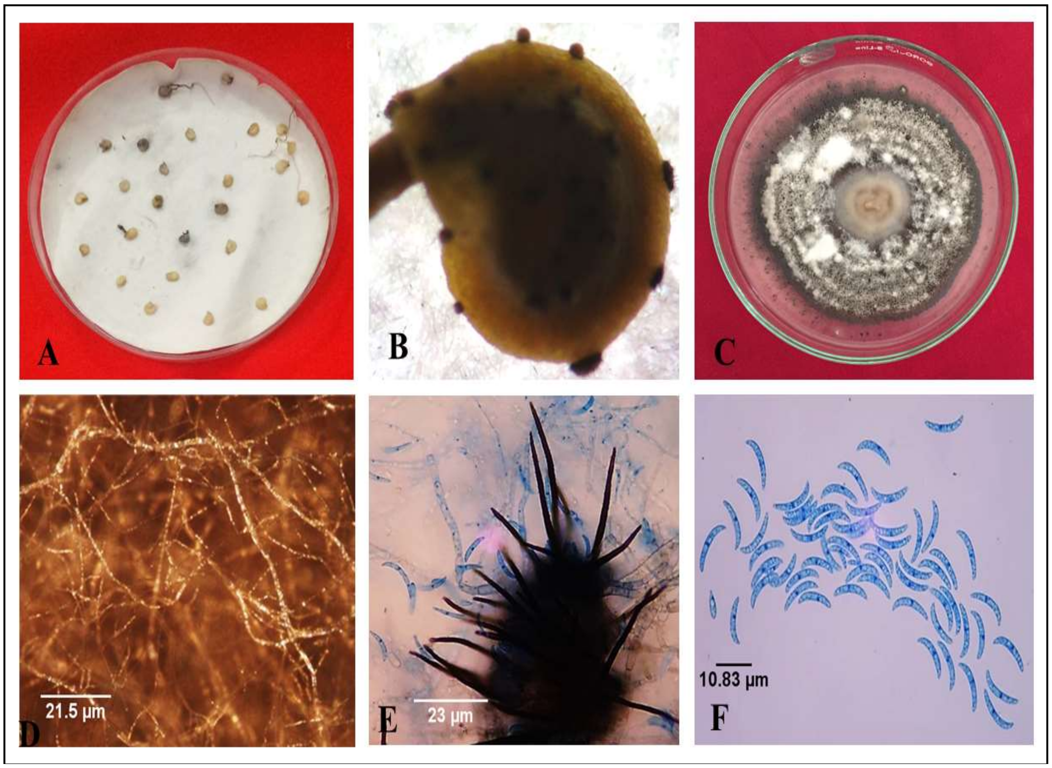

Figure 1. Isolation and identification of *C. capsici.* A - Colonization of *C. capsici* on seed; B - Habit character of *C. capsici* on seed; C - Pure culture of *C. capsici* on PDA; D- Mycelia of *C. capsici* under stereo microscope; E- Ascervuli with setae; F- Conidial structure of *C. capsici*.

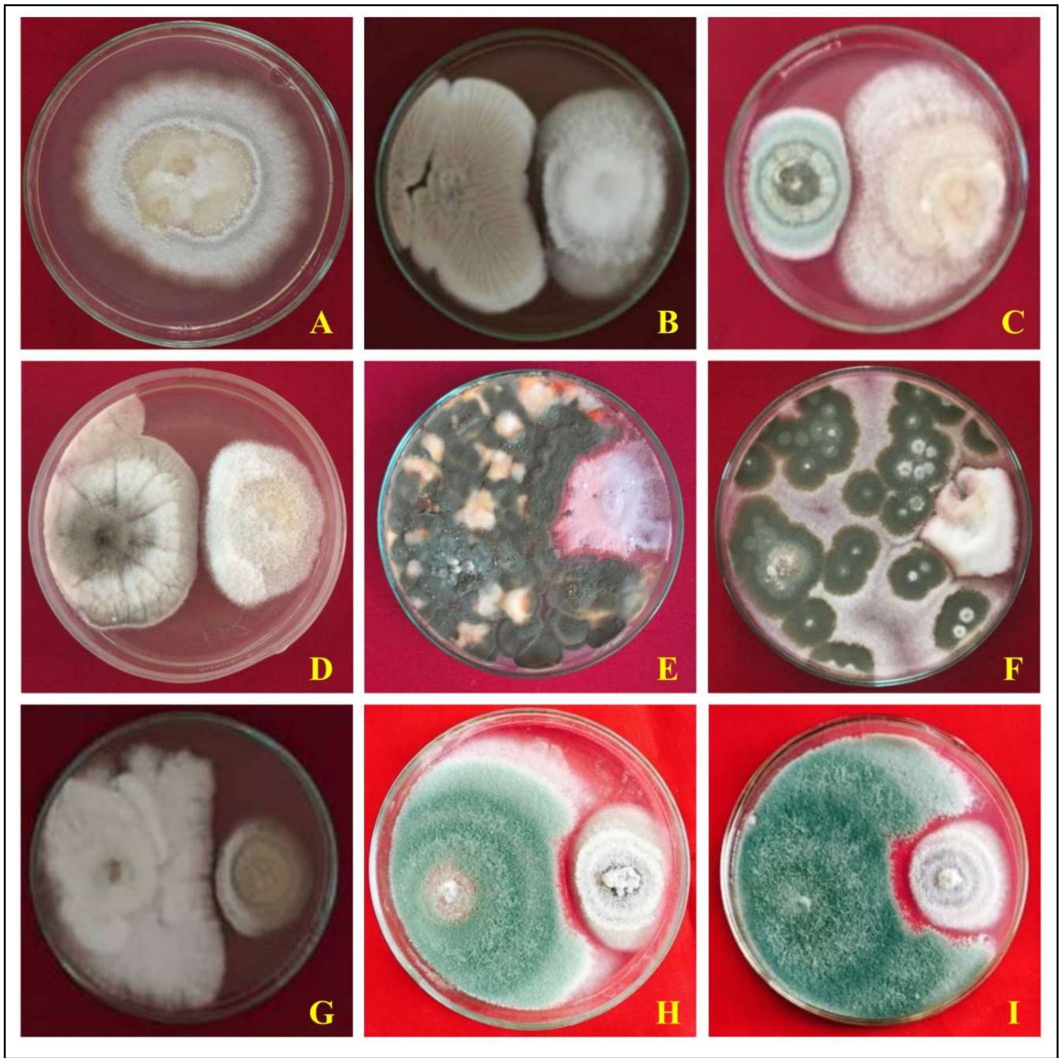

Figure 2. Antagonistic activity of PGPF against *C. capsici*. A - Control; B - NBP-08; C - NBP-22; D - NBP-44; E - NBP-45; F - NBP-61; G - NBP-65; H - NBP-66; I - NBP-67.

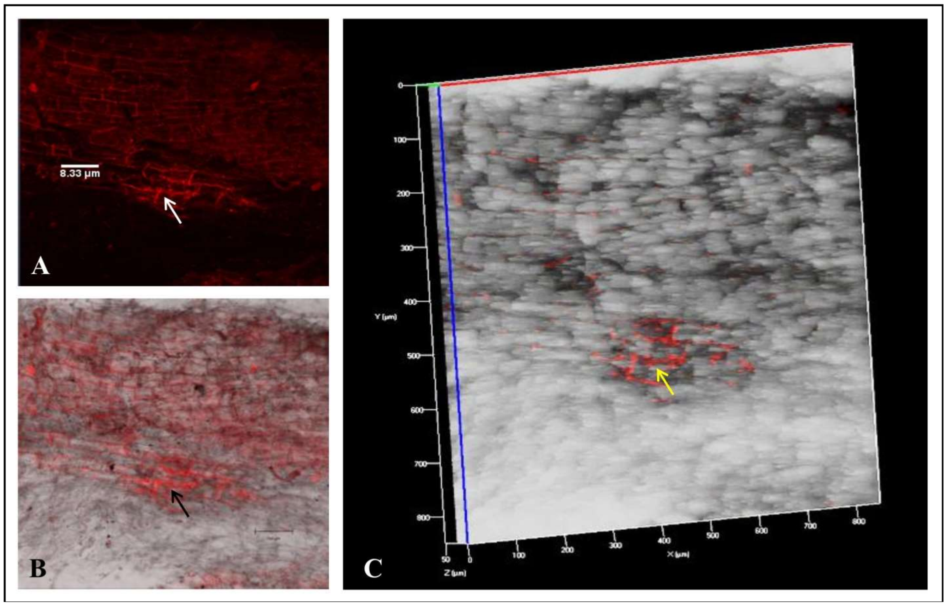

Figure 3. Representative image of root colonization ability of PGPF on chilli roots observed under Confocal microscope. A and B- PGPF colonization captured at two different fields of confocal microscope; C - 3-Dimensional image showing PGPF root colonization.

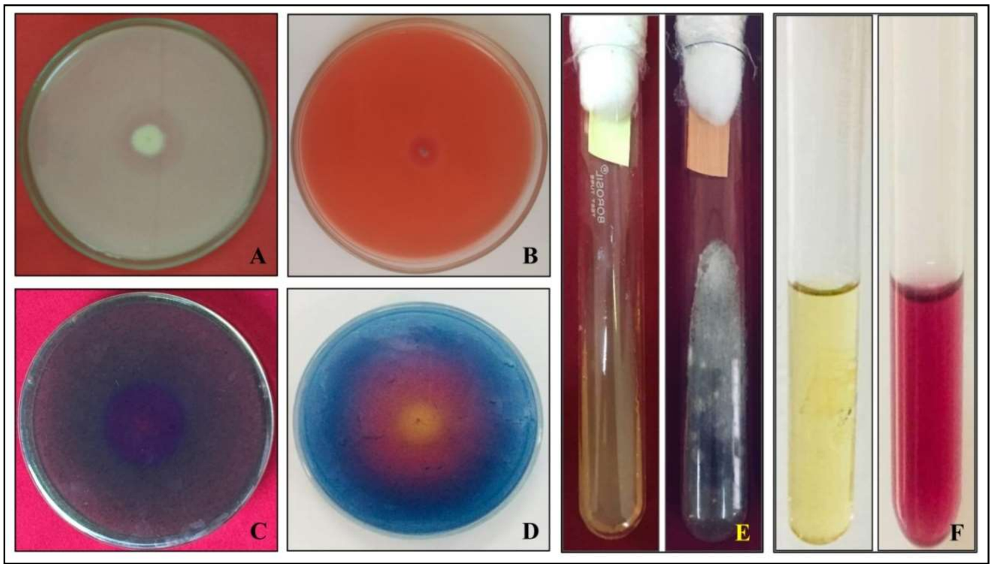

Figure S4. Representative images for plant growth promoting traits of antagonistic rhizosphere fungi. A - Phosphate solubilization; B - Cellulase production; C - Chitinase production; D - Siderophore production; E - HCN production; F - IAA production.

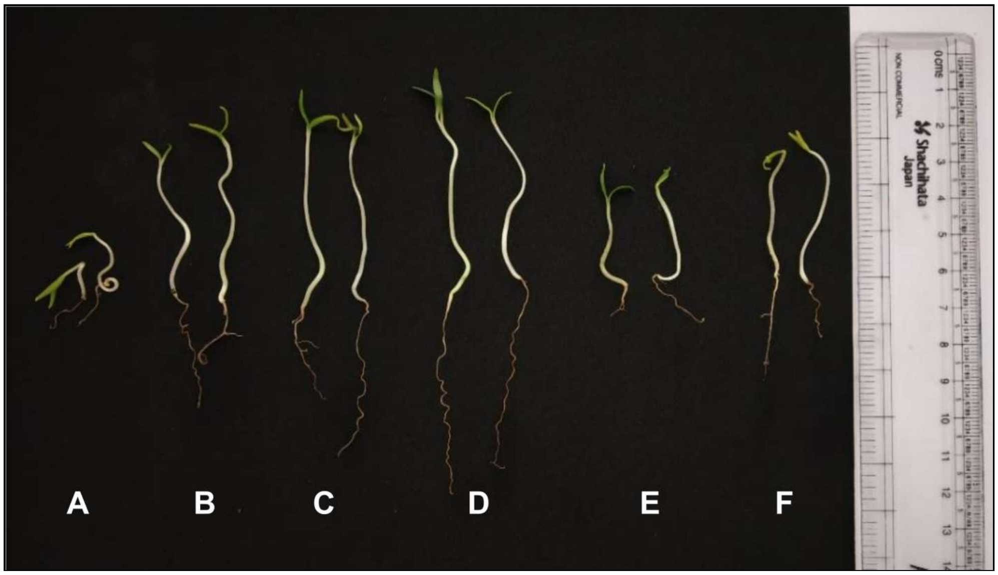

Figure S5. Effect of PGPF seed treatment on seedling vigour of chilli under *in vitro* conditions. A - Control; B - NBP-08; C - NBP-45; D - NBP-61; E - NBP-66; F - NBP-67.

Table S1. GenBank Accession Numbers of selected PGPF and pathogen.

Isolates Code	Name of the isolate	Strain	Accession No.	BLAST Closest match	Identity (%)
Aspergillus sp. NBP-08	*Aspergillus tubingensis*	**UOM PGPF 05**	MH714878	*Aspergillus tubingensis* NBMold2012B02 (KM115137)<br>*Aspergillus tubingensis* NA-TL10 (MF599714)<br>*Aspergillus tubingensis* 14R-2-F04 (KX958024)	100<br>97.93<br>97.93
Penicillium sp. NBP-45	*Penicillium* sp.	**UOM PGPF 02**	MH701853	*Penicillium* sp. LH01 (GU390692)<br>*Penicillium* sp. S6 (FJ042514)<br>*Eurotiales* sp. Rco025 (KP963597)	100<br>99.81<br>99.62
Talaromyces sp. NBP-61	*Talaromyces funiculosus*	**UOM PGPF 04**	MH701855	*Talaromyces funiculosus* M4 (MG748629)<br>*Talaromyces funiculosus* PfN23C01(MK952571)<br>*Talaromyces purpureogenus* G72(MN206956)	99.08<br>99.08<br>99.08
Trichoderma sp. NBP-66	*Trichoderma harzianum*	**UOM PGPF 03**	MN306150	*Trichoderma harzianum* CTCCSJ-A-SD3109 (KT314301)<br>*Trichoderma* sp. SDAS203463 (MK870661)<br>*Trichoderma* sp. yi1509_1 (MH284589)	100<br>98.96<br>98.79
Trichoderma sp. NBP-67	*Trichoderma asperellum*	**UOM PGPF 01**	MH490888	*Trichoderma asperellum* TR044 (KC993074)<br>*Trichoderma asperellum* BHU216 (JN604837)<br>*Trichoderma asperellum* IIPRMLKP (MK855326)	99.64<br>99.64<br>99.64
Colletotrichum sp. UOM NBP-01	*Colletotrichum capsici*	**UOM NBP 1**	MH703532	*Colletotrichum capsici* JL-6 (JX867217)<br>*Colletotrichum capsici* C-III-3 (EF016302)<br>*Colletotrichum capsici* JLCC 35348 (KF725627)	100<br>99.62<br>99.61

Table S2. *In vitro* Antagonistic nature of rhizospheric fungi against *C. capsici.*

Isolate Code	Growth Inhibition (%)	Isolate Code	Growth Inhibition (%)
NBP-01	NA	NBP-36	NA
NBP-02	NA	NBP-37	NA
NBP-03	NA	NBP-38	NA
NBP-04	NA	NBP-39	NA
NBP-05	NA	NBP-40	NA
NBP-06	NA	NBP-41	NA
NBP-07	NA	NBP-42	NA
NBP-08	77.91 ± 1.89^{cd}	NBP-43	NA
NBP-09	NA	NBP-44	58.35 ± 1.62^{e}
NBP-10	NA	NBP-45	85.48 ± 0.63^{ab}
NBP-11	NA	NBP-46	NA
NBP-12	NA	NBP-47	NA
NBP-13	NA	NBP-48	NA
NBP-14	NA	NBP-49	NA
NBP-15	NA	NBP-50	NA
NBP-16	NA	NBP-51	NA
NBP-17	NA	NBP-52	NA
NBP-18	NA	NBP-53	NA
NBP-19	NA	NBP-54	NA
NBP-20	NA	NBP-55	NA
NBP-21	NA	NBP-56	NA
NBP-22	74.13 ± 0.63^{d}	NBP-57	NA
NBP-23	NA	NBP-58	NA
NBP-24	NA	NBP-59	NA
NBP-25	NA	NBP-60	NA
NBP-26	NA	NBP-61	88.64 ± 0.72^{a}
NBP-27	NA	NBP-62	NA
NBP-28	NA	NBP-63	NA
NBP-29	NA	NBP-64	NA
NBP-30	NA	NBP-65	51.41 ± 1.20^{f}
NBP-31	NA	NBP-66	79.81 ± 1.03^{c}
NBP-32	NA	NBP-67	82.33 ± 1.03^{bc}
NBP-33	NA	NBP-68	NA
NBP-34	NA	NBP-69	NA
NBP-35	NA	NBP-70	NA

Values are means of four independent replicates (n=4) and ± indicate standard errors. Mean values followed by the same letter(s) within the same column are not significantly ($p \leq 0.05$) different according to Tukey's HSD. NA: Not Antagonistic.

Table S3. Temporal pattern accumulation of lignin deposition in chilli seedlings upon treatment with PGPF.

Treatments	Hours after post inoculation (h.p.i.)						
	0	3	6	9	12	18	24
**SU**	00.00 ±0.00^{e}	00.00 ± 0.00^{f}	02.66 ± 0.33^{f}	06.00 ± 1.15^{g}	15.66 ± 1.45^{f}	16.00 ± 0.57^{g}	21.21 ± 0.62^{g}
**SI**	00.00 ± 0.00^{e}	01.00 ± 0.00^{f}	04.00 ± 0.57^{f}	13.33 ± 0.88^{f}	18.66 ± 0.88^{f}	24.33 ± 0.88^{f}	29.55 ± 1.00^{f}
**NBP-08**	06.00 ± 0.57^{c}	07.33 ± 0.33^{c}	18.00 ± 0.57^{c}	31.00 ± 0.57^{c}	44.33 ± 0.33^{c}	52.66 ± 0.88^{c}	60.56 ± 1.14^{c}
**NBP-45**	08.00 ± 0.57^{b}	10.00 ± 0.57^{b}	22.00 ± 0.57^{b}	36.66 ± 0.33^{b}	53.66 ± 0.88^{b}	60.00 ± 1.15^{b}	66.35 ± 0.97^{b}
**NBP-61**	10.33 ± 0.33^{a}	12.33 ± 0.33^{a}	29.66 ± 0.88^{a}	44.66 ± 0.33^{a}	59.33 ± 0.66^{a}	65.33 ± 0.88^{a}	73.21 ± 1.07^{a}
**NBP-66**	01.66 ± 0.33^{e}	03.00 ± 0.57^{e}	07.33 ± 0.88^{e}	17.66 ± 0.88^{e}	24.33 ± 0.88^{e}	34.66 ± 0.88^{e}	40.27 ± 1.11^{e}
**NBP-67**	03.66 ± 0.33^{d}	05.33 ± 0.33^{d}	14.66 ± 0.33^{d}	27.00 ± 0.57^{d}	35.66 ± 0.33^{d}	44.33 ± 1.45^{d}	50.46±1.45^{d}

Values are means of three independent replicates (n=3) and ± indicate standard errors. Mean values followed by the same letter(s) within the same column are not significantly ($p \leq 0.05$) different according to Tukey's HSD. SU: Susceptible uninoculated; SI: Susceptible inoculated.

Table S4. Temporal pattern accumulation of callose deposition in chilli seedlings upon treatment with PGPF.

**Treatments**	**Hours after post inoculation (h.p.i.)**						
	**0**	**3**	**6**	**9**	**12**	**18**	**24**
**SU**	00.00 ± 00.00^{e}	00.00 ± 00.00^{f}	0.66 ± 0.33^{f}	03.00 ± 0.57^{g}	07.66 ± 0.33^{g}	13.00 ± 0.57^{g}	23.13 ± 1.08^{g}
**SI**	00.00 ± 00.00^{e}	00.00 ± 00.00^{f}	01.33± 0.33^{f}	12.66 ± 0.88^{f}	20.00 ± 1.15^{f}	25.00 ± 1.15^{f}	32.11 ± 0.99^{f}
**NBP-08**	06.66 ± 0.33^{c}	07.66 ± 0.33^{c}	18.33± 0.88^{c}	34.66 ± 1.20^{c}	43.66 ± 0.88^{c}	56.33± 0.88^{c}	61.47 ± 0.62^{c}
**NBP-45**	10.33 ± 0.88^{b}	12.00 ± 0.57^{b}	25.00 ± 1.15^{b}	40.66± 0.33^{b}	51.00 ± 0.57^{b}	61.33± 1.20^{b}	67.47 ± 0.99^{b}
**NBP-61**	14.00 ± 0.57^{a}	16.00 ± 0.57^{a}	30.00 ± 0.57^{a}	52.00 ± 0.57^{a}	64.66 ± 0.88^{a}	67.00 ± 1.15^{a}	74.23 ± 1.14^{a}
**NBP-66**	00.66 ± 0.33^{e}	02.33 ± 0.33^{e}	06.66± 0.88^{e}	23.66 ± 0.88^{e}	28.33 ± 0.88^{e}	43.66± 0.88^{e}	46.48 ± 1.80^{e}
**NBP-67**	03.33 ± 0.33^{d}	04.33 ± 0.33^{d}	14.00 ± 0.57^{d}	29.66 ± 0.88^{d}	39.00 ± 0.57^{d}	49.66 ± 0.88^{d}	53.88 ± 0.88^{d}

Values are means of three independent replicates (n=3) and ± indicate standard errors. Mean values followed by the same letter(s) within the same column are not significantly ($p \leq 0.05$) different according to Tukey's HSD. SU: Susceptible uninoculated; SI: Susceptible inoculated.

Table S5. Temporal pattern accumulation of PAL enzyme activity in chilli seedlings upon treatment with PGPF.

**Treatments**	**Hours after post inoculation (h.p.i.)**						
	**0**	**3**	**6**	**12**	**24**	**48**	**72**
**SU**	7.13 ± 0.86^{f}	08.38 ± 0.90^{f}	11.47 ± 0.84^{f}	17.23 ± 1.10^{g}	19.05 ± 0.55^{g}	30.15 ± 0.97^{g}	22.61 ± 0.46^{g}
**SI**	9.13 ± 0.69^{f}	10.45 ± 0.20^{f}	14.23 ± 1.38^{f}	22.39 ± 0.44^{f}	25.16 ± 0.36^{f}	35.04 ± 0.84^{f}	28.15 ± 0.88^{f}
**NBP-08**	18.64 ± 0.28^{c}	21.01 ± 0.24^{c}	28.74 ± 0.31^{c}	37.11 ± 0.70^{c}	43.04 ± 0.34^{c}	64.22 ± 0.32^{c}	55.10 ± 0.26^{c}
**NBP-45**	21.76 ± 0.52^{b}	24.39 ± 0.15^{b}	32.91 ± 0.72^{b}	42.35 ± 0.67^{b}	49.37 ± 0.69^{b}	69.27 ± 1.12^{b}	60.12 ± 0.93^{b}
**NBP-61**	24.98 ± 0.91^{a}	27.49 ± 0.82^{a}	37.53 ± 0.34^{a}	47.30 ± 0.69^{a}	55.76 ± 0.82^{a}	74.35 ± 0.79^{a}	65.33 ± 0.71^{a}
**NBP-66**	12.32 ± 0.20^{e}	14.13 ± 0.71^{e}	20.27 ± 0.23^{e}	27.10 ± 0.24^{e}	31.25 ± 0.21^{e}	54.06 ± 0.99^{e}	45.28 ± 0.57^{e}
**NBP-67**	15.54 ± 0.49^{d}	17.32 ± 0.64^{d}	24.59 ± 0.58^{d}	32.20 ± 0.59^{d}	37.10 ± 0.92^{d}	59.39 ± 0.93^{d}	50.37 ± 0.08^{d}

Values are means of three independent replicates (n=3) and ± indicate standard errors. Mean values followed by the same letter(s) within the same column are not significantly ($p \leq 0.05$) different according to Tukey's HSD. SU: Susceptible uninoculated; SI: Susceptible inoculated.

Table S6. Temporal pattern accumulation of POX enzyme activity in chilli seedlings upon treatment with PGPF.

Treatments	Hours after post inoculation (h.p.i.)						
	0	3	6	12	24	48	72
**SU**	02.16 ± 0.51^{f}	04.26 ± 0.09^{g}	6.27 ± 0.37^{f}	08.08 ± 0.24^{g}	11.03 ± 0.65^{g}	17.91 ± 0.74^{g}	12.75 ± 0.48^{f}
**SI**	04.57 ± 0.40^{f}	07.20 ± 0.21^{f}	8.30 ± 0.90^{f}	11.94 ± 0.30^{f}	15.45 ± 0.47^{f}	22.52 ± 0.89^{f}	14.22 ± 0.51^{f}
**NBP-08**	14.09 ± 0.49^{c}	17.46 ± 0.44^{c}	20.75 ± 0.31^{c}	24.46 ± 0.69^{c}	28.07 ± 0.59^{c}	36.94 ± 0.32^{c}	27.99± 0.92^{c}
**NBP-45**	17.53 ± 0.66^{b}	20.03 ± 0.80^{b}	23.88 ± 0.26^{b}	27.23 ± 0.16^{b}	32.26 ± 0.45^{b}	41.02 ± 0.94^{b}	32.20 ± 0.59^{b}
**NBP-61**	20.61 ± 0.74^{a}	23.46 ± 0.64^{a}	27.23 ± 0.98^{a}	30.09 ± 0.22^{a}	36.72 ± 0.72^{a}	45.06 ± 0.31^{a}	36.57 ± 0.99^{a}
**NBP-66**	07.65 ± 0.60^{e}	11.13 ± 0.37^{e}	14.26 ± 0.75^{e}	18.98 ± 0.48^{e}	20.10 ± 1.16^{e}	28.38 ± 1.05^{e}	19.22 ± 0.02^{e}
**NBP-67**	10.80 ± 0.78^{d}	14.13 ± 0.34^{d}	17.59 ± 0.33^{d}	21.11 ± 0.36^{d}	24.18 ± 0.77^{d}	32.77 ± 0.62^{d}	23.52 ± 0.58^{d}

Values are means of three independent replicates (n=3) and ± indicate standard errors. Mean values followed by the same letter(s) within the same column are not significantly ($p \leq 0.05$) different according to Tukey's HSD. SU: Susceptible uninoculated; SI: Susceptible inoculated.

Table S7. Temporal pattern accumulation of β-1,3-glucanase enzyme activity in chilli seedlings upon treatment with PGPF.

Treatments	Hours after post inoculation (h.p.i.)						
	0	3	6	12	24	48	72
**SU**	0.51 ± 0.03^{f}	01.25 ± 0.12^{g}	01.81 ± 0.01^{g}	02.97 ± 0.55^{f}	04.43 ± 0.31^{g}	06.90 ± 0.22^{g}	05.57 ± 0.26^{g}
**SI**	0.68 ± 0.03^{f}	02.33 ± 0.05^{f}	02.85 ± 0.05^{f}	04.88 ± 0.13^{f}	06.74 ± 0.42^{f}	10.47 ± 0.34^{f}	08.45 ± 0.29^{f}
**NBP-08**	03.66 ± 0.31^{c}	05.97 ± 0.09^{c}	06.13 ± 0.15^{c}	14.41 ± 0.32^{c}	15.85 ± 0.20^{c}	19.26 ± 0.60^{c}	17.20 ± 0.75^{c}
**NBP-45**	04.80 ± 0.03^{b}	07.14 ± 0.15^{b}	08.39 ± 0.06^{b}	16.75 ± 0.31^{b}	18.83 ± 0.31^{b}	22.42 ± 0.30^{b}	20.75 ± 0.28^{b}
**NBP-61**	05.87 ± 0.03^{a}	08.27 ± 0.13^{a}	10.82 ± 0.42^{a}	19.45 ± 0.71^{a}	21.72 ± 0.64^{a}	25.64 ± 0.28^{a}	23.29 ± 0.68^{a}
**NBP-66**	01.59 ± 0.24^{e}	03.54 ± 0.23^{e}	03.96 ± 0.31^{e}	08.03 ± 0.10^{e}	09.82 ± 0.30^{e}	13.48 ± 0.23^{e}	10.89 ± 0.42^{e}
**NBP-67**	02.65 ± 0.20^{d}	04.93 ± 0.20^{d}	05.04 ± 0.08^{d}	11.45 ± 0.34^{d}	13.62 ± 0.27^{d}	15.55 ± 0.66^{d}	13.88 ± 0.46^{d}

Values are means of three independent replicates (n=3) and ± indicate standard errors. Mean values followed by the same letter(s) within the same column are not significantly ($p \leq 0.05$) different according to Tukey's HSD. SU: Susceptible uninoculated; SI: Susceptible inoculated.

Table S8. Temporal pattern accumulation of chitinase enzyme activity in chilli seedlings upon treatment with PGPF.

Treatments	Hours after post inoculation (h.p.i.)						
	0	3	6	12	24	48	72
**SU**	0.20 ± 0.05[g]	0.27 ± 0.02[f]	0.45 ± 0.03[g]	1.14 ± 0.09[g]	1.55 ± 0.09[g]	2.63 ± 0.05[g]	2.99 ± 0.05[g]
**SI**	0.40 ± 0.03[f]	0.43 ± 0.02[f]	0.85 ± 0.04[f]	1.52 ± 0.06[f]	1.85 ± 0.03[f]	3.24 ± 0.08[f]	4.32 ± 0.03[f]
**NBP-08**	1.10 ± 0.00[c]	1.79 ± 0.05[c]	2.90 ± 0.06[c]	3.17 ± 0.08[c]	4.17 ± 0.02[c]	5.23 ± 0.05[c]	6.28 ± 0.03[c]
**NBP-45**	1.27 ± 0.01[b]	2.22 ± 0.02[b]	3.54 ± 0.03[b]	3.70 ± 0.08[b]	4.46 ± 0.01[b]	5.88 ± 0.06[b]	6.82 ± 0.10[b]
**NBP-61**	1.46 ± 0.01[a]	2.52 ± 0.10[a]	3.97 ± 0.05[a]	4.26 ± 0.05[a]	4.85 ± 0.05[a]	6.41± 0.04[a]	7.43 ± 0.13[a]
**NBP-66**	0.65 ± 0.04[e]	0.98 ± 0.07[e]	2.13 ± 0.04[e]	2.34 ± 0.05[e]	3.57 ± 0.02[e]	3.89 ± 0.04[e]	5.20 ± 0.13[e]
**NBP-67**	0.88 ± 0.04[d]	1.39 ± 0.05[d]	2.56 ± 0.08[d]	2.82 ± 0.05[d]	3.88 ± 0.09[d]	4.62 ± 0.11[d]	5.75± 0.11[d]

Values are means of three independent replicates (n = 3) and ± indicate standard errors. Mean values followed by the same letter(s) within the same column are not significantly ($p \leq 0.05$) different according to Tukey's HSD. SU: Susceptible uninoculated; SI: Susceptible inoculated.
